# Mathematical concepts in Arabic calligraphy: The proportions of the ʾAlif

**DOI:** 10.1371/journal.pone.0232641

**Published:** 2020-05-28

**Authors:** Mohammad Ali Jalal Yaghan

**Affiliations:** Department of Design and Visual Communication, School of Architecture and Built Environment, German Jordanian University, Amman, Jordan; University of Aveiro, NEW ZEALAND

## Abstract

The starting point of every study on the proportions of Arabic calligraphy is the letter ʾAlif. It is considered the reference for all other letters. Usually, it is measured in dots. This paper is an attempt to study the mathematical concepts upon which the historical theory of the ʾAlif proportions was based, though not mathematically stated. In order to achieve this, the terms and components of the theory were clearly defined, and analyzed in their textual and visual context, historically, and logically according to our present time. In spite of the frequent use of these terms throughout time, their meanings were not always clear and can accept different interpretations. Some terms, even, indicated different meanings and were loosely used to satisfy different functions, for example, the term “Nuqṭah” (dot). Relating the components of the theory was, also, opened for opinions and interpretations, for example, the number of dots for the ʾAlif was never agreed upon. This paper starts with historical research and analysis. Then, it presents the mathematical expressions of the ʾAlif proportions both numerically and visually. Later, it discusses how they do apply, how other modern interpretations can fit, and how historical misunderstandings can be understood. Finally, it presents the historical account of how to relate other letters to the ʾAlif recommending analyzing their mathematical aspects in future studies.

## Introduction and definitions

Arabic calligraphers used a reed pen sharpened in a certain way resulting in a writing nib that is a short dash. This gave variety in thickness for different strokes depending on the angle it is placed with and on the hand movement. Using this pen, the calligraphers created so many scripts overtime along many paths of evolution.

As a major milestone, calligraphers defined a theory of how to relate all letters to the first letter (the ʾAlif), for which they dedicated the first special part of the theory. This theory was stated textually (though leaving a lot to interpret and induce), and visually both in the manuscripts about calligraphy or other manuscripts as sample writings (again with a space for interpretations).

Modern studies attempted to analyze the theory textually and visually, but the mathematical analysis is scarce. This study is an attempt to study the first part of the theory, which is the proportions of the letter ʾAlif, historically and mathematically.

Many studies on Arabic calligraphy applied the notion of metrics found in calligraphy manuals on different aspects of the Arabic digital typography. For example, Bayar and Sami discussed the basis for designing a dynamic font that applies a dynamic stretching for characters and the use of vertical and horizontal ligatures according to the Calligraphic rules [1]. Benatia, Elyaakoubi, and Lazrek showed how the classical algorithms of text justification must be revised to be able to accommodate for the cursive nature of Arabic writing and the rules found in calligraphy manuals [2]. The purpose of these studies is to extend the application of historical rules of Arabic calligraphy, found in the manuals of famous calligraphers, to modern typography.

Although the current research also applies the notion of metrics, its novelty stems from the fact that it discusses the mathematics and the proportions of the historical data itself, which represents the sources of these manuals and rules; rather than applying it.

The following are definitions for some terms that will be used throughout the paper.

The Qalam (pen): a part of a reed, cut and prepared (sharpened) as the tool for writing Arabic Calligraphy.

Placing angle: Pen nib positioning angle measured from a horizontal line.

Al-ʾIʿjām: Distinguishing between identical-shaped letters by adding dots [3, p. 50]

Al-Shakl: placing the diacritic marks that indicate the short vowels, and proper vocalization of Arabic letters [4, p. 9].

Al-Naqṭ “Dotting”: putting, writing, or drawing dots within the writing. Historically, it served both Al-ʾIʿjām and Al-Shakl according to the role of the dot at the time. Abū Bakr al Sarrāj (d. 928) explained that using dots distinguishes between similar letters, for example, the Bā, Tā, and Thā [4, p. 9]. In writing early Qurans, however, “al-Naqṭ” was used to indicate the short vowels as explained by Abu Bakr Al-Dāni (d. 1053) [5, p. 4] and was, later, replaced by the current standard Shakl diacritic marks. Currently, the first definition is the only standard.

## Scope definition and methodology

The proposition of the theory of proportions in Arabic calligraphy is simple; the ʾAlif letter is drawn according to some criteria and is measured by a number of dots in order to achieve a whole-number proportion. Then other letters are referenced either to the ʾAlif or, in a later historical stage, to the ʾAlif along with the measuring dots. The purpose of this research is to express this proposition mathematically and visually, study resulting possibilities, and relate them to the theory.

### Methodology

1. A literature review of historical documents, modern research and calligraphy manuals that refer to or utilizes the topic of proportions. Many such works provide valuable textual data, while some provide it in a visual manner.
2. Providing proper and accurate definitions of the theory and of its components, and exploring all possibilities of their scopes.
3. Expressing the defined relations mathematically.
4. Building tables of possible values for all variables
5. Visually representing the ʾAlifs of all the values and comparing them.
6. Discussing results, drawing conclusions, and explaining other approaches suggested by current researches.

## The proportions of the letter ʾAlif

The letter ʾAlif is the first letter in the Arabic Alphabet. In all references about Arabic calligraphy, the starting point is always the ʾAlif and the surrounding circle with the measuring unit being the dot [6, p. 131; 7, pp. 27–38; 8, p. 70; 9, p. 35; and 10, p. 195] (footnote1).

This system of proportions is usually attributed to Ibn Muqla (d. 940) who is an iconic figure in Arabic calligraphy. He was a wazīr (minister) for three Abbasid califs, and a reputable calligrapher [10, pp. 457–160]. Al-Tawḥīdī (d. 1023), even, quoted a contemporary calligrapher describing Ibn Muqla as a prophet of calligraphy [11, p. 37].

Most of the calligraphers and writers, who followed, presented Ibn Muqla as the founder of the Arabic calligraphy proportional system (in what they refer to as al-Khaṭṭ al-Mansūb) and the creator of the standardizer of most of the famous calligraphic styles [7, p. 17; 8, p. 46; and 12, p. 15]. The second issue was doubted by some historical studies [8, p. 38] and challenged by many modern [9, p. 33]. Alain George, who provided an introduction of the concept of proportions in Islamic artistic aspects and how it relates to Greek thought [13, pp. 95–114], even refuted the authoritative status of Ibn Muqla as the one to introduce proportions into the calligraphy field, and suggested it was there before him [13, pp. 134–137] (footnote 2). Blair suggested that the work of Ibn Muqla was about a style that she referred to as “broken cursive” (an intermediate stage between the book round script and the standardized round script by the later Ibn al-Bawwāb (d. 1022)) [10, pp. 143–178].

Nevertheless, the analytical study presented here concentrates on the proportions of the letter ʾAlif as defined by the work contributed to Ibn Muqla and many later writers who claimed to quote him and extended his theory. First, its shape will be introduced, and then its proportions.

## The ʾAlif shape

Ibn Muqla, and all writers that follow, like al-Qalqashandī (d. 1418), defined the ʾAlif as a vertical line that is not slanted to the right nor to the left.

"قال الوزير أبو علي بن مقله: و هي [الألف] شكل مركب من خط منتصب، يحب أن يكون مستقيما غير مائل إلى استلقاء ولا انكباب، قال: و ليست مناسبةً لحرف في طول ولا قصر."

“The waziīr ʾAbu ʿAli Ibn Muqla said: and it [the ʾAlif] is a shape composed from a vertical line that should be straight, not slanted to the right (ʾistilqāʾ) nor to the left (ʾinkibāb), he said: and it is not proportioned to any letter in tallness or shortness.” [7, p. 27].

The condition for the ʾAlif to be correct as stated by Ibn Muqla is:

"… واعتبارها أن تخط إلى جانبها ثلاث ألفات أو أربع ألفات فتجد فضاء ما بينها متساوياً."

“Its criteria [of correctness] is to write to its side three ʾAlifs or four ʾAlifs and find space in-between equal.” [7, p. 28].

**Fig 1:**
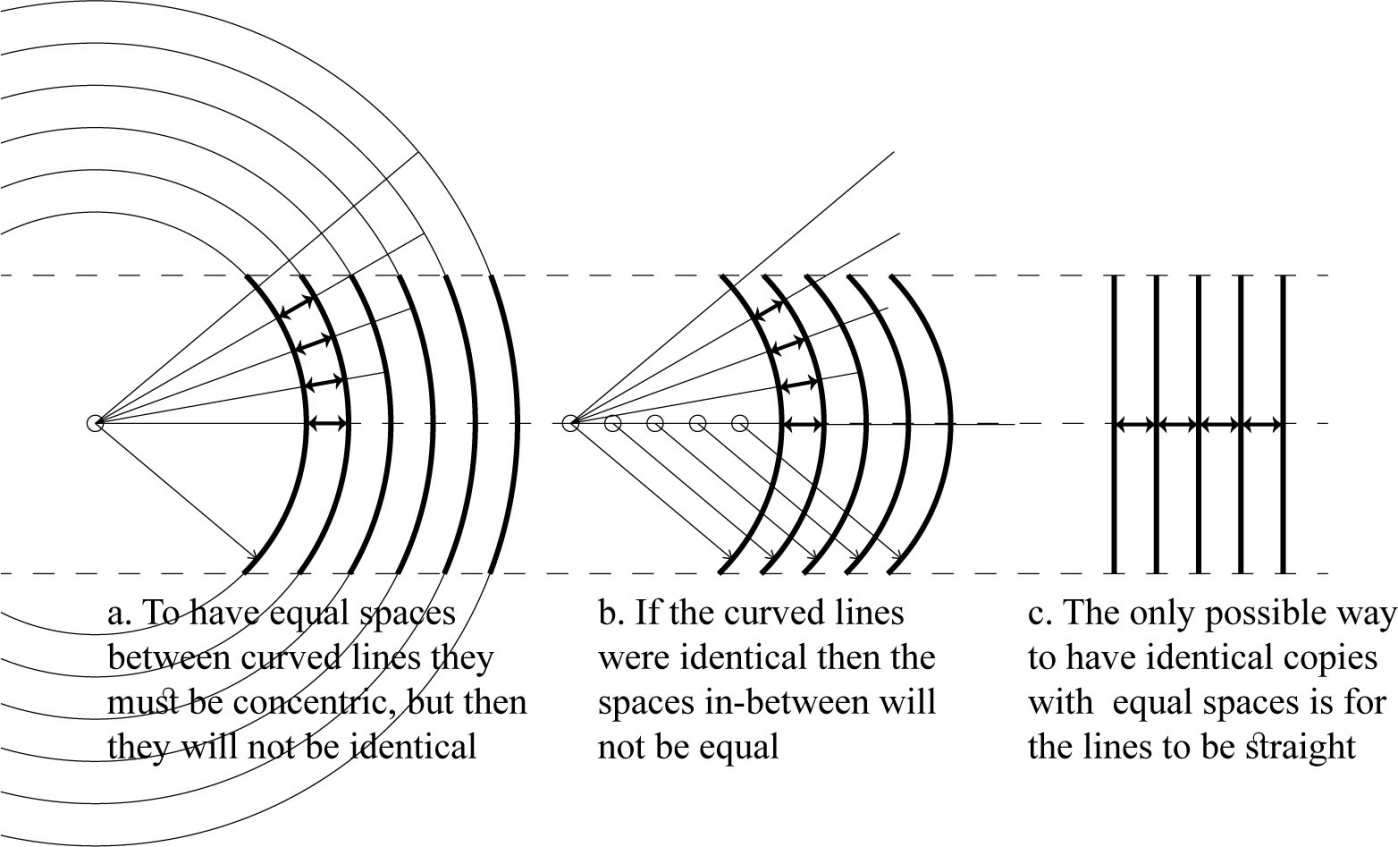
Concentric circles of different sizes result in equal spaces in-between, while a repeated curve results in unequal spaces.

This condition is a very clever guarantee of the straightness of the ʾAlif. It seems to be stated by someone who is clearly profound in geometry, but refrained from discussing the geometrical and mathematical aspects, and giving simple practical rules for calligraphers to follow. Any curvature in the shape of the ʾAlif would result in unequal spaces between its identical copies as they will not be concentric, and the spaces in-between will, thus, vary in width (Fig 1).

Some might argue that the term فضاء ما بينهما (“space in-between”) introduced by Ibn Muqla refers to the horizontal distance between every two horizontally aligned points, and this is the equality required by his criterion-condition. If this was the case, then the condition will not discriminate any shape repeated at equal horizontal shifts as this will always result in equal horizontal distances, and, thus, it will be meaningless.

The author’s understanding is that the term “space in-between” refers to the whole space in-between the shapes, which should be equally even, equally offset, and unified. It is not a reference to the horizontal distances only. As Fig 1A shows, to achieve equal spaces in-between curvy shapes requires them to be concentric (or equally offset), in which case, they will not be identical. On the other hand, as Fig 1B shows, to repeat identical curvy shapes at equal horizontal distances will disturb the “spaces in between” making them unequal and uneven. Only a repeated straight line, Fig 1C, can achieve the condition of “equal space in-between.” Ibn Muqla introduced his ʾAlif as a straight line, and then proposed his criterion-condition as the measure to guarantee it’s straightness. This understanding of Ibn Muqla’s work is the means to enable his condition to achieve its purpose and to be a valid meaningful condition.

Ikhwān al-ṣafā (fl. late 10^th^ early 11^th^ c.) confirmed the verticality of the ʾAlif.

"ينبغي أن يكون صور الحروف كلها لأي أمة كانت في أي لغة كانت وبأي أقلام خطت إلى التقويس و الإنحنا ما يكون إلا الألف الذي في كتابة العربية…"

“All the images of the letters, for whichever nation, in whichever language, and in whichever pens written with, should be to curving and bending whatever they should except for the ʾAlif in writing Arabic” [14, p. 98]

Later writers, always started their work by such a definition for the ʾAlif. For example Ibn al-Ṣāʾigh (d. 1441) [8, p. 68], and later ʿAbdullāh ibn ʿAlī Al-Hītī, (d. 1486), who wrote:

"…الألف حرف منتصب القامة من غير ميل ولا اعوجاج ليس له شبيه بالحروف…"

“The ʾAlif is a letter that has a vertical height without slanting or curvature, it has no parallel between letters…” [15, p. 418]

Even a much later calligrapher Al-Ṣaydāwi (fl. between the 15^th^ and the 18^th^ c. as suggested in [16, p. 438]) who authored a poem about calligraphy (”Waḍḍaāḥat al-ʾUṣūl fi al-Khatt”) also started talking about the ʾAlif defining it as vertical, straight, and upright [16, p. 449].

The vertical ʾAlifs were also the subject of praise for calligraphers. Al-Tawḥīdī (d. 1023), for example, quoted Ibn al-Musharraf al-Baghdādī who claimed that he saw the writings of Aḥmad ibn Abī Khālid, the writer for the Calif al-Maʾmūn, presented by the “king of the Rūm” (Byzantine Emperor) for his people as part of his decorations in the days of celebration. He quoted that its ʾAlifs and Lāms were at their utmost in verticality and straightness [11, p. 36].

However, this definition of a vertical ʾAlif, does not apply to most of the historical styles, whose ʾAlifs were not exactly vertical. Nevertheless, writers kept on introducing the ʾAlif as such at the beginning of any discussion, then they would move to the ʾAlifs of specific scripts.

This discrepancy between the theory provided by the writers and the actual application might be justified by the explanation provided by Ikhwān al Ṣafā:

"وهذا الذي ذكرناه من نسب هذه الحروف و كمية مقادير أطوالها بعضها عند بعض هو بوجه قوانين الهندسة و النسب الفاضلة فأما ما يتعارفه الناس و يستحسنه الكتاب فعلى غير ما ذكرنا من المقادير والنسب وذلك بحسب موضوعاتهم و اختياراتهم دون غيرها وبحسب طول الدربة وجريان العادة فيها"

“And this, what we mentioned of the proportions of these letters and the relations of their lengths to each other, is what proposed by the rules of geometry and the virtuous ratios. As for what people are accustomed to, and what writers prefer, they are of different values and proportions according to their subjects, own choices, and according to the length of their experience and their habits.” [14, p. 98]

However, there are some actual occurrences of vertical ʾAlifs. For example, the papyri of Qurrah Ibn Shatīk (d. 96 H), which contained mainly slanted ʾAlifs, contained also vertical ones [17, p. 116, and table of page 321]. Another example was presented by Shella Blair when she was talking about some manuscripts with “later marginal ascriptions attributing the transcription to Ibn Muqla.” She asserts that they are a good example of broken cursive and have a straight vertical line for the ʾAlif [10, p. 159 and figure 5.7].

Alain George, and after presenting a treatise dated to 959 with the ʾAlifs appearing as straight lines [13, p. 127, figure 74], stated that:

“In several cursive manuscripts dated between 969 and 993, the letters do begin to tend towards the straight line and the circle, yet there is no stylization of the strokes along geometrical lines.” [13, p. 127]

**Fig 2:**
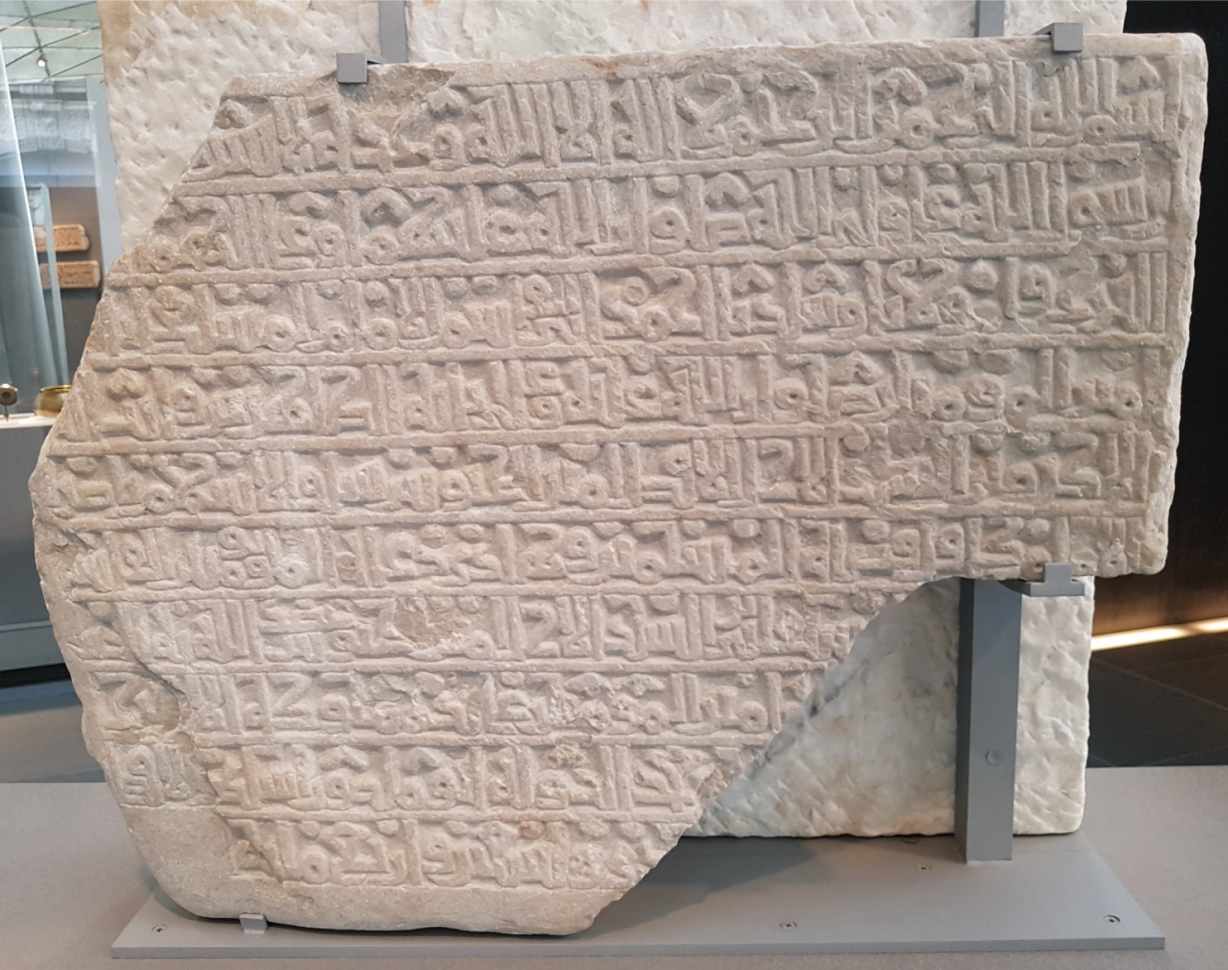
An example of using a straight ʾAlif, 1097 foundation inscription of the Fatimid wazīr al-Afdal, Lebanon-Sayda, currently in the Louvre.

We also see the ʾAlif as a vertical line in the works of Ibn al-Bawwāb (d. 1022) [10, p. 163, figure 5.8; and 13, p. 129, figure 76], and in some foundation scripts and stelas of the era (Fig 2).

Of the contemporary styles of our times, the ʾAlif in the riqʿa style is a straight line but inclined to the left.

The actual existence of few examples the ʾAlif as defined by Ibn Muqla, the use of the above definition as the starting point in the historical works on calligraphy, and the emphases of these works on the ʾAlif as being the origin and the reference for all other letters, all led this study to consider the ʾAlif as a vertical line, and study its proportions as such. First, we will introduce the measuring unit; the dot.

## The dot

**Fig 3:**
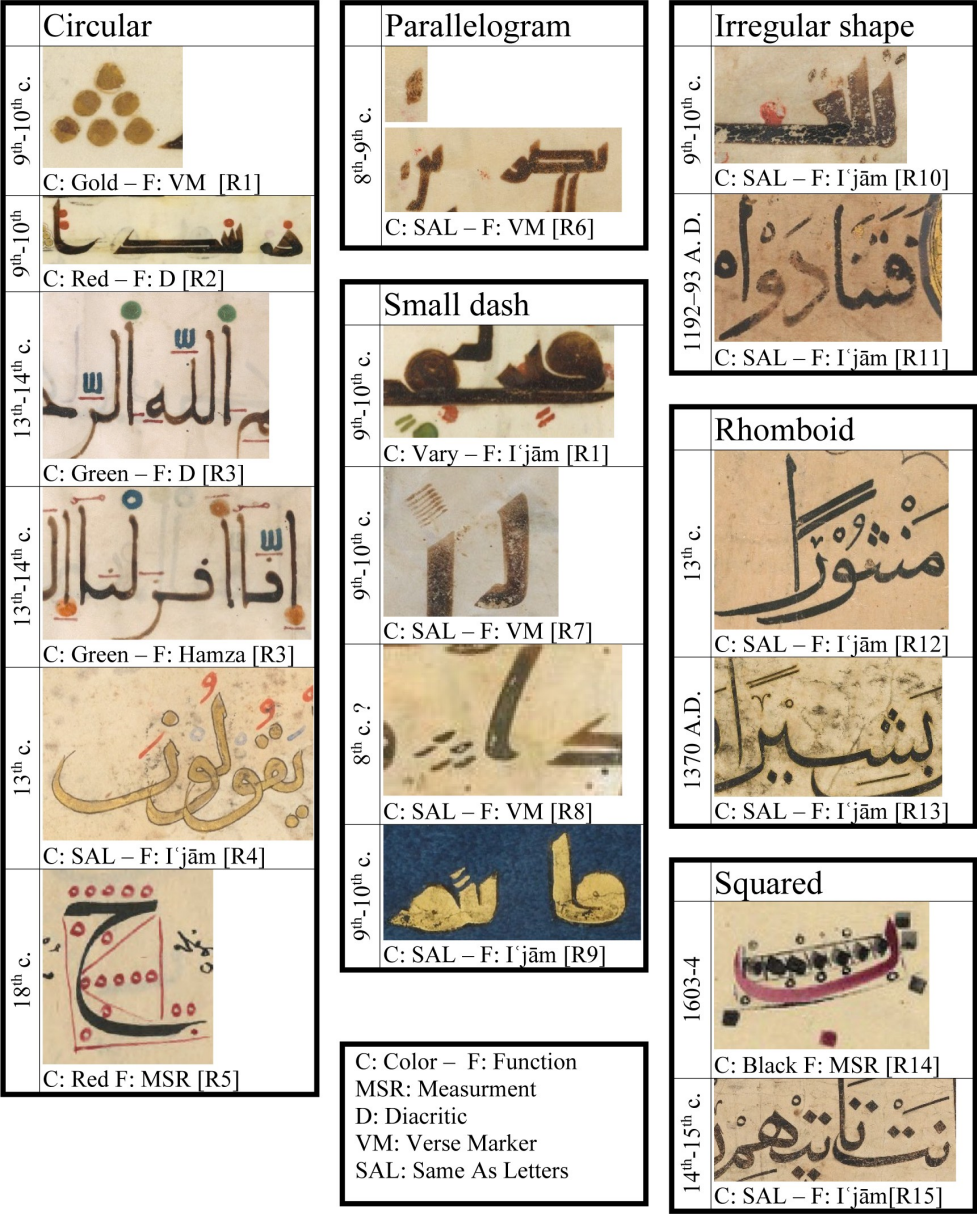
The shapes and functions of the dot in Arabic writing.

Over time, the dot (Arabic: Nuqṭah) had many usages and functions in the context of Arabic calligraphy and layout. It was used as a verse marker in the Quran (as single or in a group), a diacritic mark, to donate the Hamza, to distinguish between similar letters “ʾIʿjām”, a measuring unit, and, only in our current time, a full stop or part of other punctuation marks. It appeared in many shapes; a circle, a parallelogram, a small dash, a square, a rhomboid, and even an irregular shape. At times it took the same color of the letters, but in many others, it was drawn in a different color to visually demonstrate the distinction of its function. For example, circular dots were, at some point, drawn in red ink, in contrast to the black letters, to denote the short vowels and the tanwīn (nunnation); and drawn in orange and in green to donate other diacritic issues. Fig 3 presents examples for all these shapes, colors and functions.

According to Al-Dāni, (d. 1053) and other historical writers, the first use of dots was to denote the ending of the verses of the Qurʾān (verse markers) [5, p. 2] Then, they were used in red color and circular shape to denote the short vowels [5, p. 6–7; and 7, pp. 160 and 164], while other colors were used to donate other vocalization issues [5, pp. 19–24]. At the same time, short strokes (Shaʿr “hair” [5, p. 22]) were used to distinguish similar-shaped letters “ʾIʿjām”. Later, the current diacritic marks were adopted and replaced the colored dots.

A final added visual function for the dot was laid in the theory of proportions where it played the role of the measuring unit for the ʾAlif [7, pp. 28–38] and later for other letter parts [8, pp. 104–105; and 18, pp. 608–617], to becoming the sole measuring unit for all letters as seen in modern calligraphic manuals [19, pp. 7–8, 13–14, 28, and 51].

The current functions of the dots are the “ʾIʿjām”, being part of the punctuation marks, and being units of measurement.

The historic role of the dot in being a measuring unit for the ʾAlif, is the concern of this paper.

## The shape of the dot as a measuring unit

In spite of the existence of the many shapes for the dot, as shown in Fig 3, Ibn Muqla and the later historical writers on proportions indicated that there are only two shapes for the dot, the circle and the square [7, p. 155; 20, p. 362]. The square was the major reference unit [7, p. 28; 8, p. 68].

Modern manuals use the dots as the measuring unit for the strokes of all letter, and while squares (or near square shapes) are the more common [19, pp. 7–8, 13–14, 28, and 51], circles are sometimes used to indicate the number of dots with open triangles for their halves [as seen in [21, pp. 36–78].

Abbott, and later Blair, however, stated that the dot is a rhomboid with its sides depending on the width of the nib [9, p. 35; and 10, pp. 159 and 211]. Some other modern manuals stated that it is a rectangle (stressing that they are with right angles) for certain types of scripts (footnote 3).

**Fig 4:**
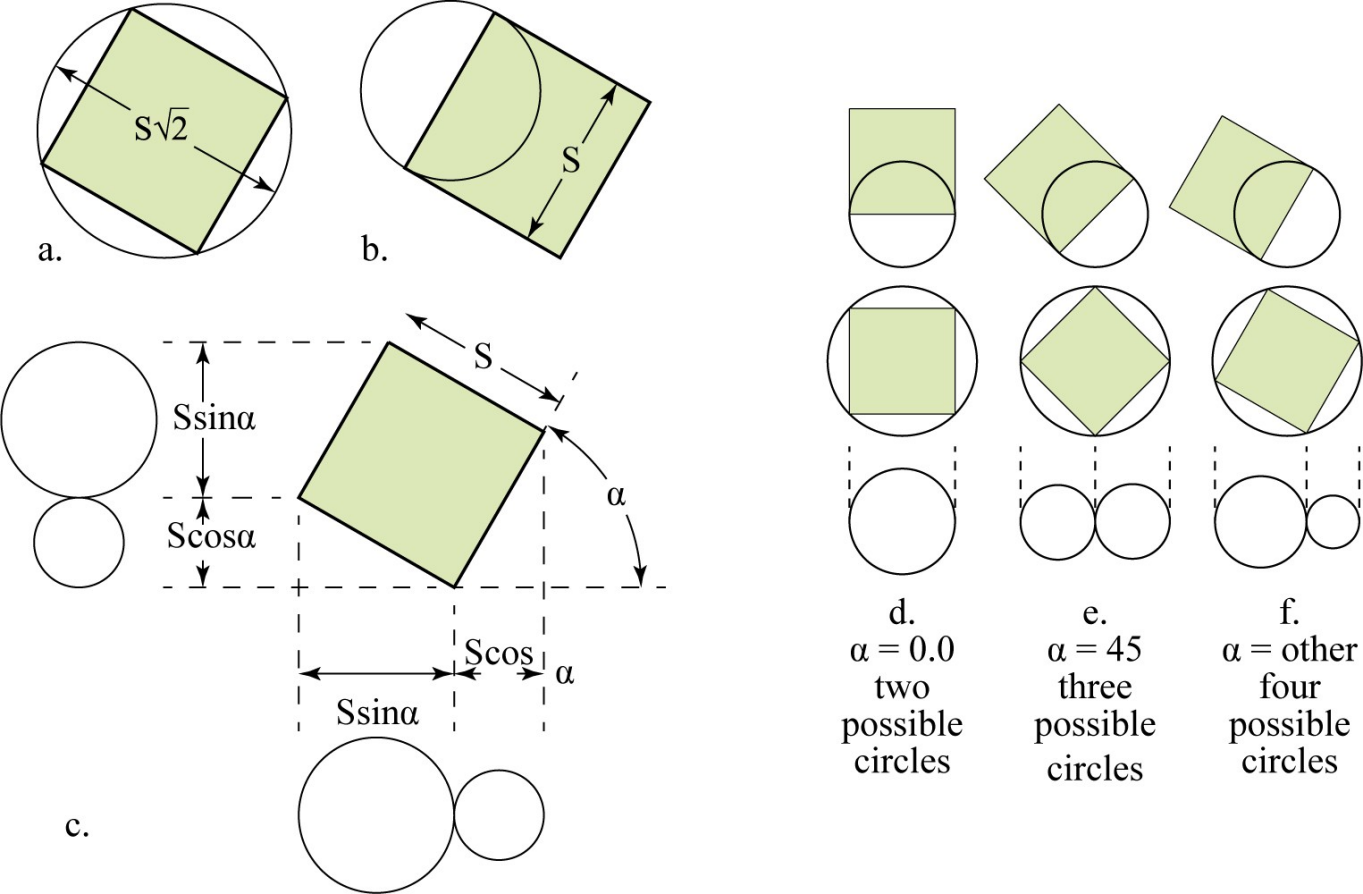
The possible sizes for a circular dot in relation to a squared one.

While the size of the squared dot is obvious, with its edge being equal to the pen nib, there are many possibilities for the radius of the circular dot. First, it could be the circle that circumference the dot with a diameter that equals the square root of two multiplied by the dot side (Fig 4A). Second, it could be the circle with a diameter that equals the side of the dot (Fig 4B). Third, it could be the circle (or circles) whose diameter is equal to the horizontal and vertical projections of the dot side depending on the placing angle (Fig 4C). The total number of possible circles could, thus, be two if the placing angle was 0° (Fig 4D), three if the placing angle was 45° (Fig 4E), or four otherwise (Fig 4F).

Historically, there is no reference to the first circle because it gives a bigger dimension than the original squared dot. The second circle is, however, referenced in the work of Ibn al-Ṣāʾigh where he states:

"… نقطة دائرة وتكون النقطة بوجه القلم…"

“… circular dot and the dot will be by the face of the pen …” [8, p. 106]

**Fig 5:**
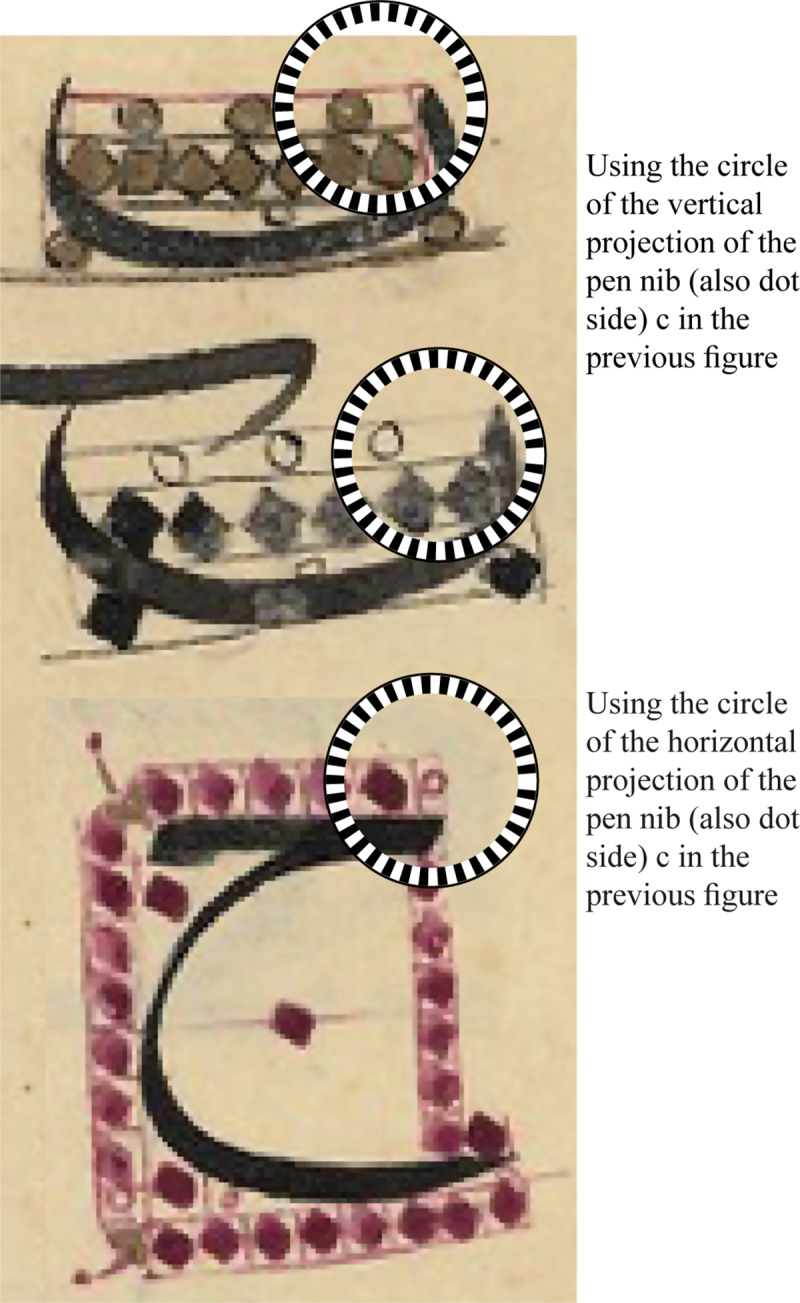
Historical examples for using the circular dots of diameters equal to the vertical and horizontal projections of dot side (which is equal to the pen nib). Photo source: parts of page 29 of [R^14^].

The third circle size can be seen in use in later historical works, as seen in a 1603–4 A.D copy of “Hüsn-ü hat risalesi” by Mehmet Ibn Tacettin (d. 1587) (Fig 5) [22, p.29].

Small circles with no geometrical relation to the dot are sometimes used as a convention to indicate the number of dots but not to actually represent them. Examples can be seen in an 18^th^-century copy of the same book of Ibn Tacettin (Fig 3—bottom cell of the first column) and in the work of Ibn al-Ṣāʾigh [8, pp. 104–105]. This convention continued to be used by many modern calligraphic manuals, for example see [21, p. 69].

This study, and since it is about the proportions of the ʾAlif in the historical context, which inform us of a clear definition of a square shape, will analyze the proportions using the squared dot, relate them to the circular shape, and will, also, propose how modern suggestions of dot shapes can achieve similar or approximate results.

## Relating the ʾAlif to the dot

Al-Qalqashandī stated that the dot was used to measure the length of the ʾAlif, as well as its proportions (length to width).

"ثم الذي ذكره صاحب "رسائل إخوان الصفا" في رسالة الموسيقى، عند ذكر حروف المعجم استطرادا أن مساحتها [الألف] في الطول تكون ثمانَ نقط من نقط القلم الذي تكتب به ليكون العرض ثُمُن الطول."

“Then, there is what the writer of Rasāʾil Ikhwān al-Ṣafā wrote in the epistle of music, when he mentioned the alphabet, that the length of the ʾAlif is eight dots of the dots of the pen they are written with, so the width will be eighth that of the length.” [7, p. 28]

He also quoted al-ʾĀthārī,

"واعلم أن صاحبنا الشيخ زين الدين شعبان الآثاري في ألفيته قد جعل طول الألف سبع نقط من كل قلم، و مقتضاه أن يكون العرض سُبُع الطول."

“Know that our friend al-shaykh Zayn al-Dīn Shaʿbān al-ʾĀthārī in is his poem made the length of the ʾAlif seven dots from every pen, which leads for the width to be seventh the length.” [7, p. 47]

Although the actual text of Ikhwān al-Ṣafā does not relate the ʾAlif to dots but talks about its proportions being one to eight [14, pp. 97–98], al-Qalqashandī, had an obvious belief that the dots do compose the ʾAlif as building units and, thus, their width would be the width of the ʾAlif and their accumulative vertical length would determine its length. Such a belief was also present in the writings of Muḥammad ibn ʿAli ibn Sulaymān Al-Rāwandī who lived in the thirteenth century after Ikhwān al-Ṣafā, and before al-Qalqashandī. When he explained how to write each letter he finished with a quatrain; that for the ʾAlif was:

"- كل طريقة يحيط بها خاطرك،

عن علم الخط تتساوى فيها هذه النكتة.

إذا وضعت بالقلم عشر نقط على الورقة،

فإنه يتكون منها جميعا خط هو الألف."

“Every method that your mind comes up with

Regarding calligraphy, has this wisecrack:

If you laid ten dots with a pen on a paper

Then from them, all will compose a line, which is the ʾAlif”

[18, p. 608]

This concept can be vailed only when the dots are places horizontally (when placing angle equals to zero). In other cases, the width of the ʾAlif would be the horizontal projection of the nib of the pen and not the whole dot. In a later section, other plausible interpretations of al-Qalqashandī and al-Rāwandī statements will be suggested.

Regarding the number of dots, Al-Qalqashandī quoted different calligraphers giving the values six, seven, and eight as the number of dots to compose the length of the ʾAlif. A generation after, ʿAbdullāh ibn ʿAlī Al-Hītī, (d. 1486) gave additional values and stressed that the ʾAlif should not exceed a certain number of dots, different for each calligraphic style. His words can also be seen to fall within the same understanding.

"…الألف حرف منتصب القامة من غير ميل ولا اعوجاج ليس له شبيه بالحروف كالأسل لا يزيد و لاينقص عن تسع نقط مثاله (شكل 1) و قيل على سبع نقط، وقيل على خمس نقط. والأول يحمل على المحقق و الثلث، و الثاني يحمل على التواقيع الثلثية، والثالث على الرقاع."

“The ʾAlif is a letter that has a vertical height without slanting or curvature, it has no parallel between letters, it does not exceed or lessen than nine dots, and it is said seven dots, and it is said five dots. The first can be considered for the Muḥaqqaq and Thuluth scripts, the second for the al-Tawāqīʿ al-Thuluthiyyah script, and the third for the Riqāʿ script.” [15, p. 418]

In the minds of the historical writers this difference in the number of dots composing the ʾAlif seems to have posed a defect they needed to justify, thus, they proposed that each number is for a certain type of script. This justification, however, was not enough for Ibn al-Ṣāʾigh, and he proposed another geometrical one:

"الألف حرف منتصب ليس له شبه في الحروف و هو متميز في الطول على ألف الثلث مقدار نقطة و ذلك لأن ألف الثلث في صدرها تحديب ما وفي عجزها كذلك وألف المحقق ليس فيها تحديب و أرباب التقويم قالو إن الخط المستقيم أقرب من الخط المحدودب فيُزاد في ألف المحقق بمقدار تفاوت التقوير في الف الثلث فتكون ألف المحقق أرشق في الطول"

“The ʾAlif is a vertical letter that has no similar between other letters, and it is peculiar in length over the ʾAlif of the Thuluth with one dot because the ʾAlif of the Thuluth has some convexity in its chest as well as in its tail, and the ʾAlif of the Muḥaqqaq does not have any. The lords of “al-taqwīm” said that the straight line is closer than the convex one, accordingly, the ʾAlif of the Muḥaqqaq should be increased by a value equal to the difference of the convexity of the ʾAlif of the Thuluth, and, thus, the ʾAlif of the Muḥaqqaq is svelter in its length.” [8, pp. 107–108]

In spite of such justifications, it is obvious that calligraphers did not agree on the “right” number of dots nor the right proportions. However, they all agreed on one basic axiom of the theory; a whole number of dots determine the length of the ʾAlif and affect its proportions. To study this mathematically, the way the dots are connected must be investigated.

## How dots are arranged

In all visual materials about dots and how they relate to the ʾAlif, a number of dots are stacked vertically on top of each other, with an ʾAlif, of equal height, drawn beside them. The way the dots are related is affected by the placing angle which will be given the value of α.

**Fig 6:**
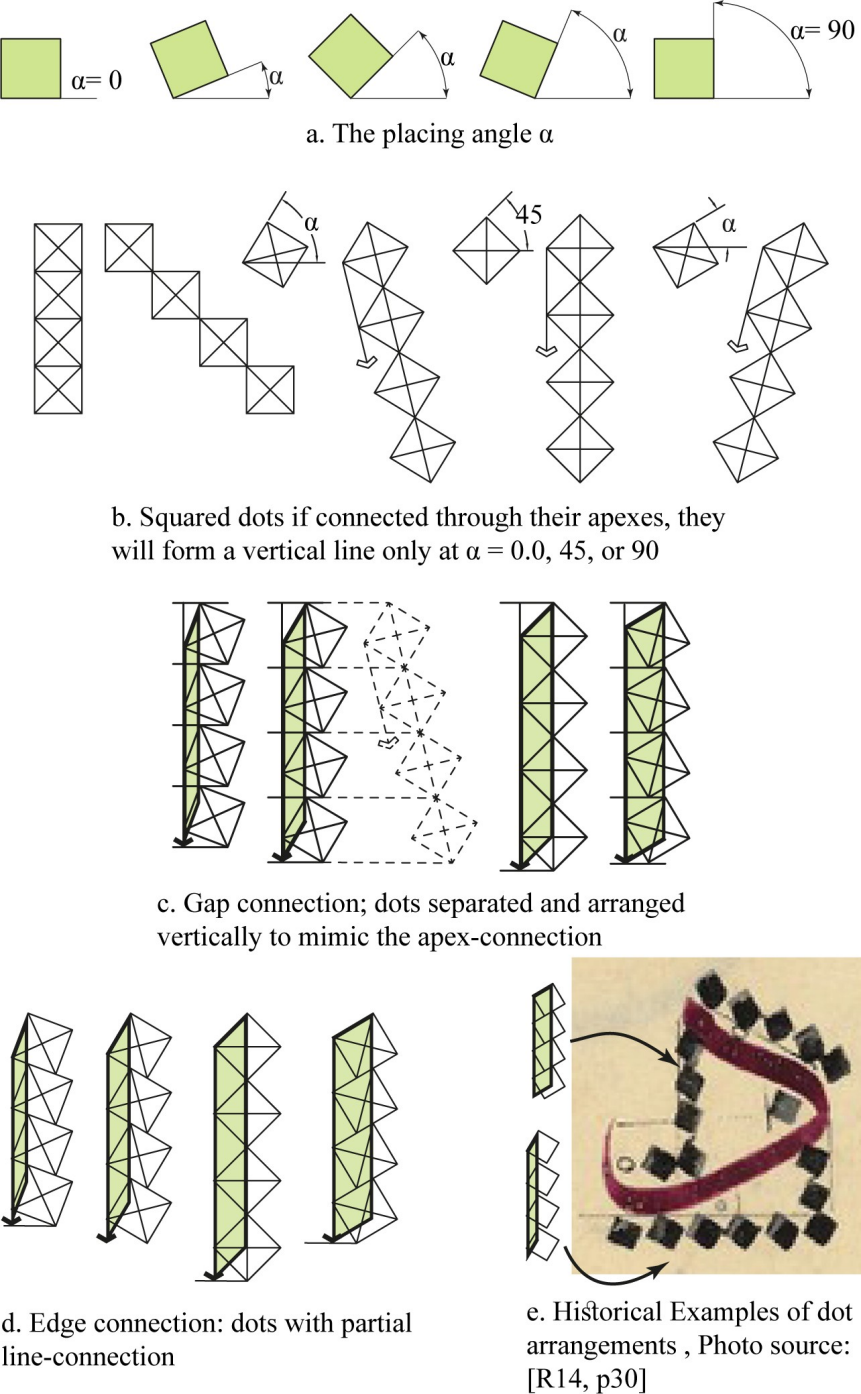
How dots are connected.

If α was 0° or 90° then the dot will be horizontal (Fig 6A), and the dots must be connected via their edges.

If the relation between dots should be a vertex to vertex [9, p. 35; and 10, p. 159], then the only placing angle to create a vertical line arrangement is 45°. All other placing angles would result in an inclined arrangement of the dots (Fig 6B).

There are two ways to achieve a vertical arrangement with other angles; the first is to arrange the dots vertically leaving gaps between them but keeping the whole dot as the vertical measuring unit (will be referred to as “gap connection”, Fig 6C). The second is to have the dots connected along their edges in a partial manner that would allow verticality according to the placing angle (will be referred to as “edge connection”, Fig 6D). Both of these two ways are plausible when attempting to interpret historical examples as shown in Fig 6E. The 0°, 45°, and 90°-instances referred to above are possible to achieve as instances of both of these two cases.

## The ʾAlif proportions

When drawing the ʾAlif, one should place the nib of the pen according to the “placing angle” and draw a vertical line. The width of the ʾAlif to its total length should be a relation of one to a whole number. Historical resources did not agree on the number itself, but all agree that is should be a whole number. Ikhwān al-Ṣafāʼ, even, considered certain relations to whole numbers as the virtuous ratio “al-nisba al-faḍila” [14, p. 98].

We will investigate the mathematical relations of the placing angle, the number of dots, and the intended proportions for both cases of dot-connection mentioned above.

### Gap connection case

**Fig 7:**
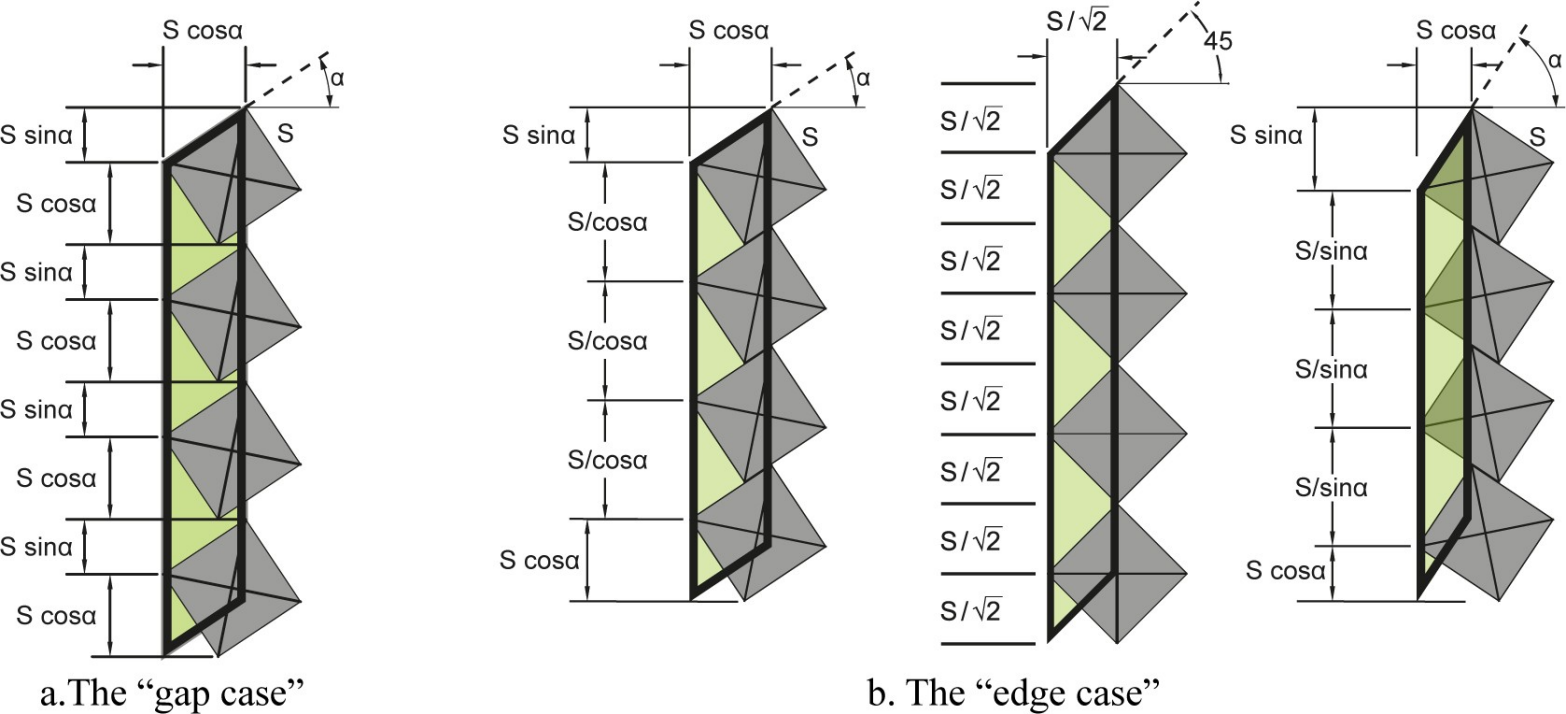
The relative dimensions of an ʾAlif in both of the “gap case” and the “edge case”, note the three possibilities in the latter.

Let the placing angle = *α*, the side of the squared dot = *S*, the number of dots to arrange = *n*, the required proportions for the ʾAlif is 1: *P*, both *n* and *P* should be whole numbers (integers), see Fig 7A.

The width of the ʾAlif = *S*×cos *α*

The length of the ʾAlif = *n*(*S*×cos *α*+*S*×sin *α*)

To get a whole number proportions:

P×S×cosα=n(S×cosα+S×sinα)

P=n(1+tanα)

Thus, the placing angle that applies the above conditions will be:

$$\tan^{-1}\alpha =\frac{P}{n}-1$$

**Fig 8:**
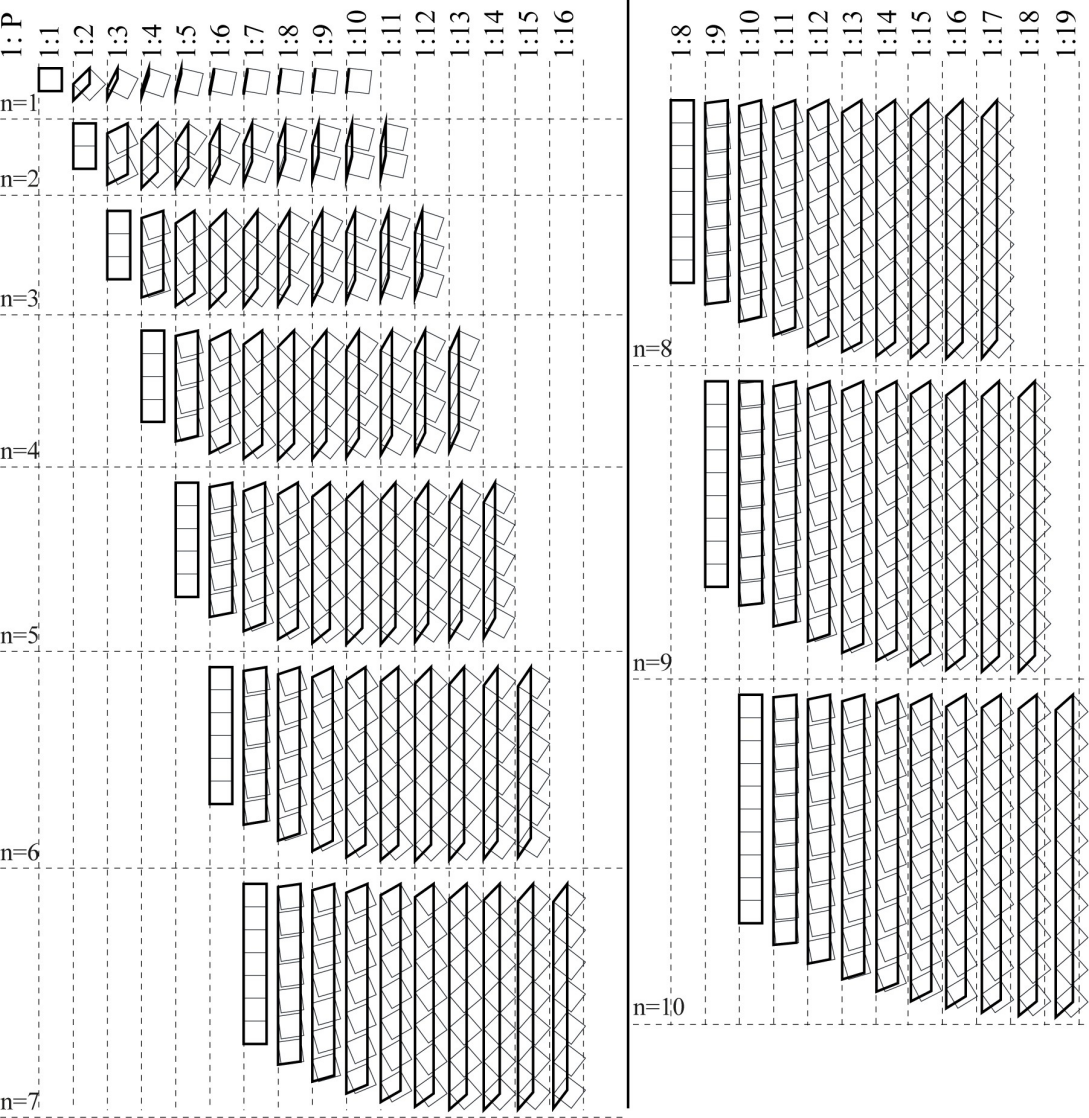
Whole number ʾAlif-proportions for the “gap case” of Table 1.

According to this formula, an arrangement of *n* dots can result in many ʾAlifs that have a whole number (*P*) proportions, depending on the placing angle value. Table 1 shows these possibilities, and Fig 8 shows their visual display.

**Table 1:** Possible values for *n (number of dots)*, *P (the desired proportions 1*: *P)*, *α (the placing angle)* that result in whole number ʾAlif proportions for the gap case.

*n* = 1		*n* = 2		*n* = 3		*n* = 4		*n* = 5	
*P*	*α*	*P*	*α*	*P*	*α*	*P*	*α*	*P*	*α*
1	0	2	0	3	0	4	0	5	0
2	45	3	26.565	4	18.435	5	14.036	6	11.31
3	63.435	4	45	5	33.69	6	26.565	7	21.801
4	71.565	5	56.31	6	45	7	36.87	8	30.964
5	75.964	6	63.435	7	53.13	8	45	9	38.66
6	78.69	7	68.199	8	59.036	9	51.34	10	45
7	80.538	8	71.565	9	63.435	10	56.31	11	50.194
8	81.87	9	74.055	10	66.801	11	60.255	12	54.462
9	82.875	10	75.964	11	69.444	12	63.435	13	57.995
10	83.66	11	77.471	12	71.565	13	66.038	14	60.945
*n* = 6		*n* = 7		*n* = 8		*n* = 9		*n* = 10	
*P*	*α*	*P*	*α*	*P*	*α*	*P*	*α*	*P*	*α*
6	0	7	0	8	0	9	0	10	0
7	9.462	8	8.13	9	7.125	10	6.34	11	5.711
8	18.435	9	15.945	10	14.036	11	12.529	12	11.31
9	26.565	10	23.199	11	20.556	12	18.435	13	16.699
10	33.69	11	29.745	12	26.565	13	23.962	14	21.801
11	39.806	12	35.538	13	32.005	14	29.055	15	26.565
12	45	13	40.601	14	36.87	15	33.69	16	30.964
13	49.399	14	45	15	41.186	16	37.875	17	34.992
14	53.13	15	48.814	16	45	17	41.634	18	38.66
15	56.31	16	52.125	17	48.366	18	45	19	41.987

### Edge connection case

Let the placing angle = *α*, the side of the squared dot = *S*, the number of dots to arrange = *n*, the required proportions for the ʾAlif is 1:*P*, both *n* and *P* should be whole numbers (integers). See Fig 7B.

If *α* = 45°, then *P* = 2 *n*

Else if *α* < 45 then

The width of the ʾAlif = *S* cos *α*

The length of the ʾAlif = *S* sin *α*+*S* cos $\alpha +\frac{S\;\left(n-1\right)}{\cos\alpha }$ To get a whole number proportions:

$$P\times (S\;\cos\propto )=S\;\sin\;\alpha +S\;\cos\;\alpha +\frac{S(n-1)}{\cos\alpha }$$

$$P\;\cos\propto =\sin\;\alpha +\cos\;\alpha +\frac{\left(n-1\right)}{\cos\alpha }$$

$$(P-1)\;\cos\propto =\sin\;\alpha +\frac{\left(n-1\right)}{\cos\alpha }$$

Else if *α* > 45 then

The width of the ʾAlif = *S* cos *α*

The length of the ʾAlif = *S* sin *α*+*S* cos $\alpha +\frac{S\;\left(n-1\right)}{\sin\propto }$

To get a whole number proportions:

$$P\times (S\;\cos\propto )=S\;\sin\;\alpha +S\;\cos\;\alpha +\frac{S(n-1)}{\sin\propto }$$

$$P\;\cos\propto =\sin\;\alpha +\cos\;\alpha +\frac{\left(n-1\right)}{\sin\propto }$$

$$(P-1)\;\cos\propto =\sin\;\alpha +\frac{\left(n-1\right)}{\sin\propto }$$

**Fig 9:**
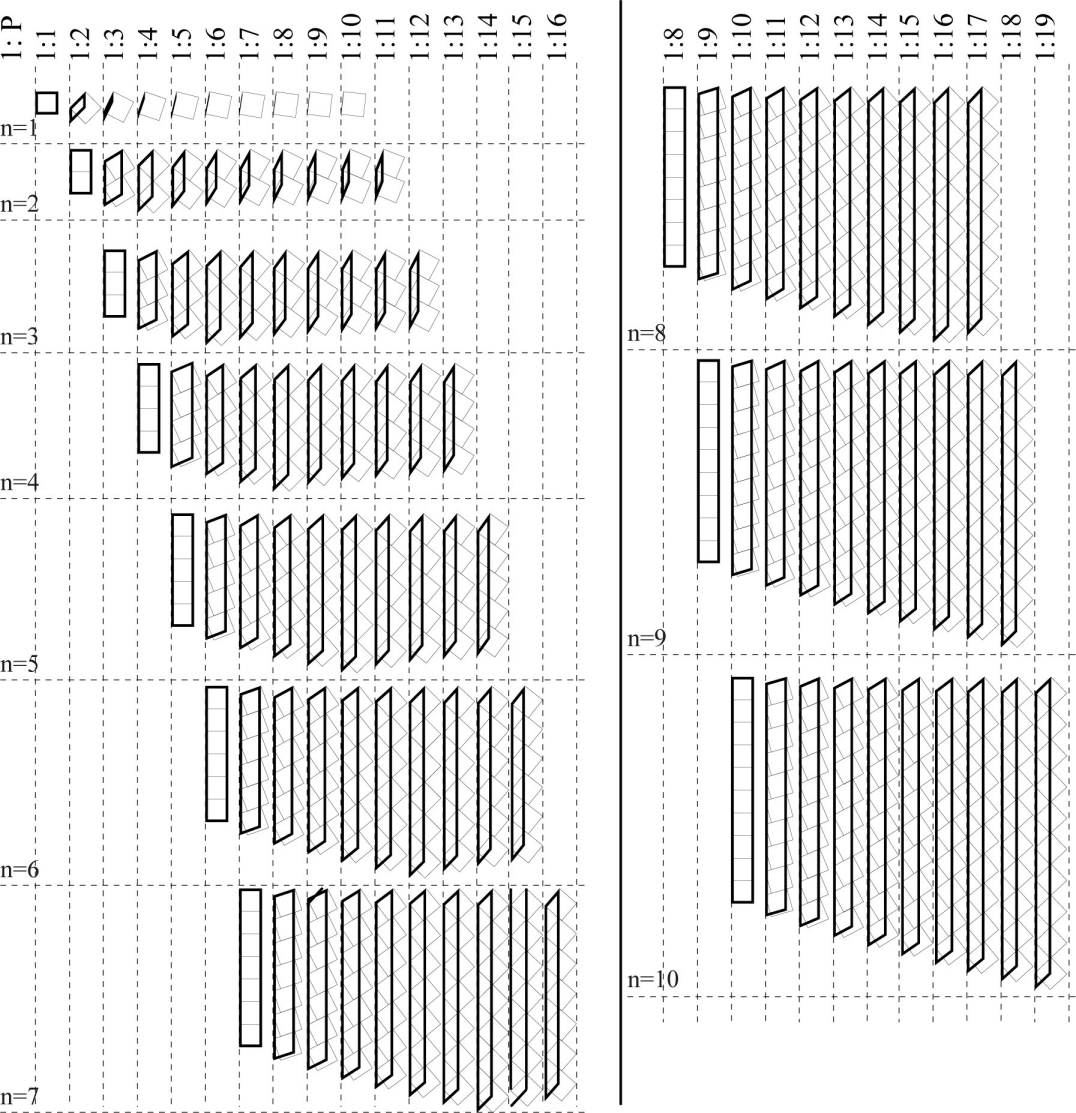
Whole number ʾAlif-proportions for the “edge case” of Table 2.

Table 2 shows the possible placing angles to get whole number proportions for each number of dots, and Fig 9 shows their visual display.

**Table 2:** Possible values for *n (number of dots)*, *P (the desired proportions 1*: *P)*, *α (the placing angle)* that result in whole number ʾAlif proportions for the edge case.

*n* = 1		*n* = 2		*n* = 3		*n* = 4		*n* = 5	
*P*	*α*	*P*	*α*	*P*	*α*	*P*	*α*	*P*	*α*
1	0	2	0	3	0	4	0	5	0
2	45	3	31.717	4	26.565	5	23.473	6	21.325
3	63.435	4	45	5	37.982	6	33.69	7	30.671
4	71.565	5	59.639	6	45	7	40.269	8	36.87
5	75.964	6	66.325	7	57.626	8	45	9	41.437
6	78.69	7	70.493	8	63.435	9	56.31	10	45
7	80.538	8	73.383	9	67.273	10	61.517	11	55.356
8	81.87	9	75.515	10	70.072	11	65.07	12	60.118
9	82.875	10	77.156	11	72.227	12	67.739	13	63.435
10	83.66	11	78.461	12	73.945	13	69.847	14	65.975
*n* = 6		*n* = 7		*n* = 8		*n* = 9		*n* = 10	
*P*	*α*	*P*	*α*	*P*	*α*	*P*	*α*	*P*	*α*
6	0	7	0	8	0	9	0	10	0
7	19.71	8	18.435	9	17.392	10	16.517	11	15.768
8	28.383	9	26.565	10	25.072	11	23.816	12	22.739
9	34.256	10	32.156	11	30.418	12	28.945	13	27.675
10	38.66	11	36.405	12	34.523	13	32.918	14	31.526
11	42.145	12	39.806	13	37.838	14	36.149	15	34.678
12	45	13	42.618	14	40.601	15	38.861	16	37.337
13	54.621	14	45	15	42.957	16	41.186	17	39.628
14	59.036	15	54.031	16	53.543	17	43.212	18	41.634
15	62.156	16	58.166	17	53.543	18	45	19	43.41

### Notes and discussion

The historical values for n or P range between 5 and 10 [15, p. 418; 7, pp. 28 and 47; and 18, p. 608], these values are emphasized in gray in the tables.

**Fig 10:**
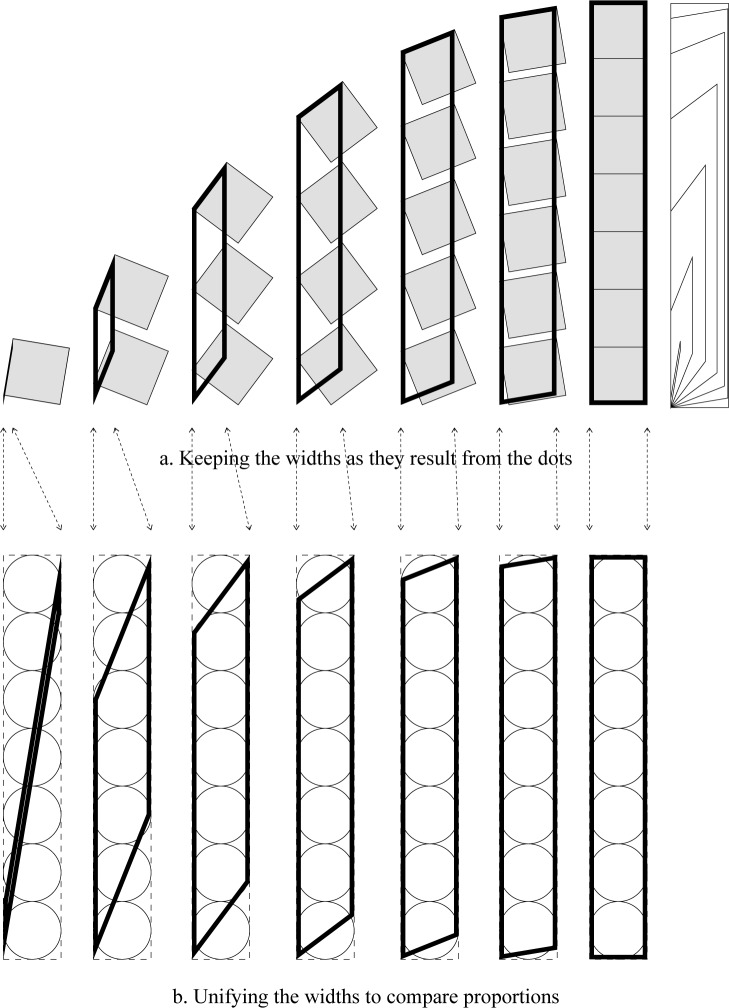
Arriving at 1:7 proportions from 1 to 7 dots.

For any *P* intended proportions, there are *P* possible values for *n* (the number of dots) to achieve such proportions. The range of n values that result in *P* proportions is *n* = [1,*P*]. The bigger the intended proportions the more the possibilities are. This applies in the two cases discussed above, so the total number of possibilities is actually double this number (Fig 10).

**Fig 11:**
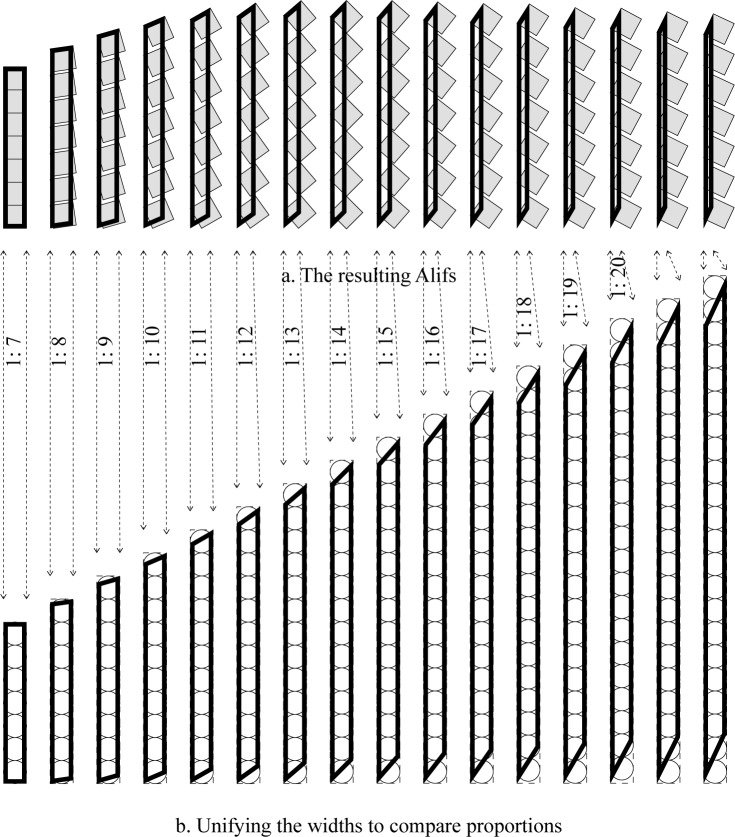
Seven dots create an endless number of whole-number possible proportions.

On the other hand, for any *n* number of points, there are endless possible values for *P* possible whole number proportions ranging from *P* = *n* to *P* = ∞, i.e. *P* = [*n*,∞]. The corresponding α values are 0.0 and 90.0 respectively. This means that when the placing angle is horizontal then the resulting proportions will equal the number of dots, while when the placing angle is 90.0 the width will be zero and the proportions will reach infinity. This is true for any *n* value (Fig 11).

**Fig 12:**
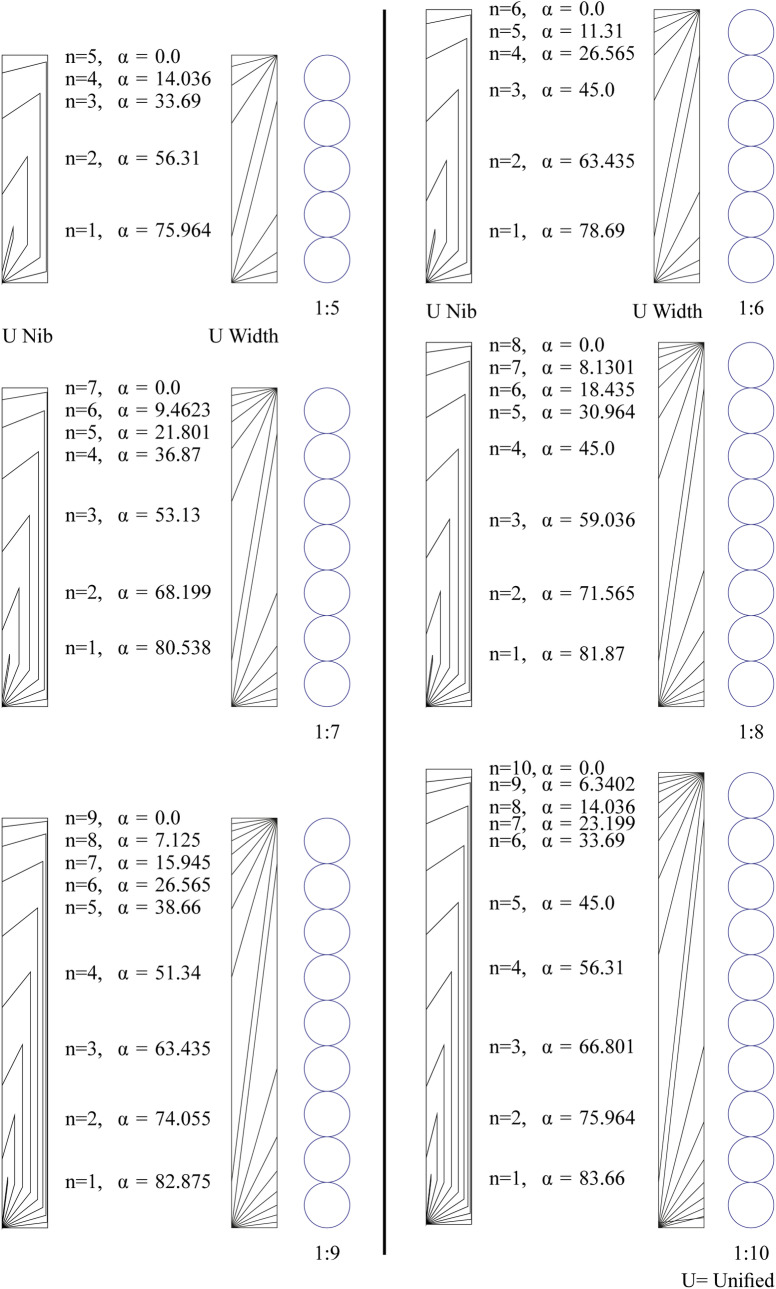
Comparing the historically common values of n and P and the corresponding α in the gap case, unifying the nib width on the left and unifying the width of the ʾAlif to the right.

Fig 12 presents a comparison of the historically common values of n and P and the corresponding α in the gap case.

These possibilities are only for a single letter (the ʾAlif), expanding the concept to other letters would mean the combinations are huge in numbers.

Such a large number of possibilities induces the following issues:

1. It Represents calligraphy as a personal reflection of any calligrapher, who can assume his or her own combination, and this would always give place for self-expression and provide variety for the audience. The role of the calligrapher is, thus, not only to follow a fixed set of rules but also to make his/ her own choice of the huge number of permutations. It might, also, explain why famous calligraphers where distinguished.
2. As most of the calligraphers were not mathematicians, or have only a little experience with mathematics, these alternative possibilities made it possible to visually write and achieve without the burden of fully comprehend the mathematics of the theory.
3. An important fact to remember is that historical calligraphy is done by hand, and was, always, at a relatively small size no matter how large a manuscript was or the writings within. These two factors (being manual and in small size) made it hard to differentiate between close values for the placing angle α. For example, check the lower values from Tables 1 and 2. This, also, gave place for personal reflections and gave a high degree of tolerance for inaccurate attempts.
4. There are still many combinations to explore even in our current times.
5. Explaining other cases of dot shapes and arrangement:

i. The rhomboid case

**Fig 13:**
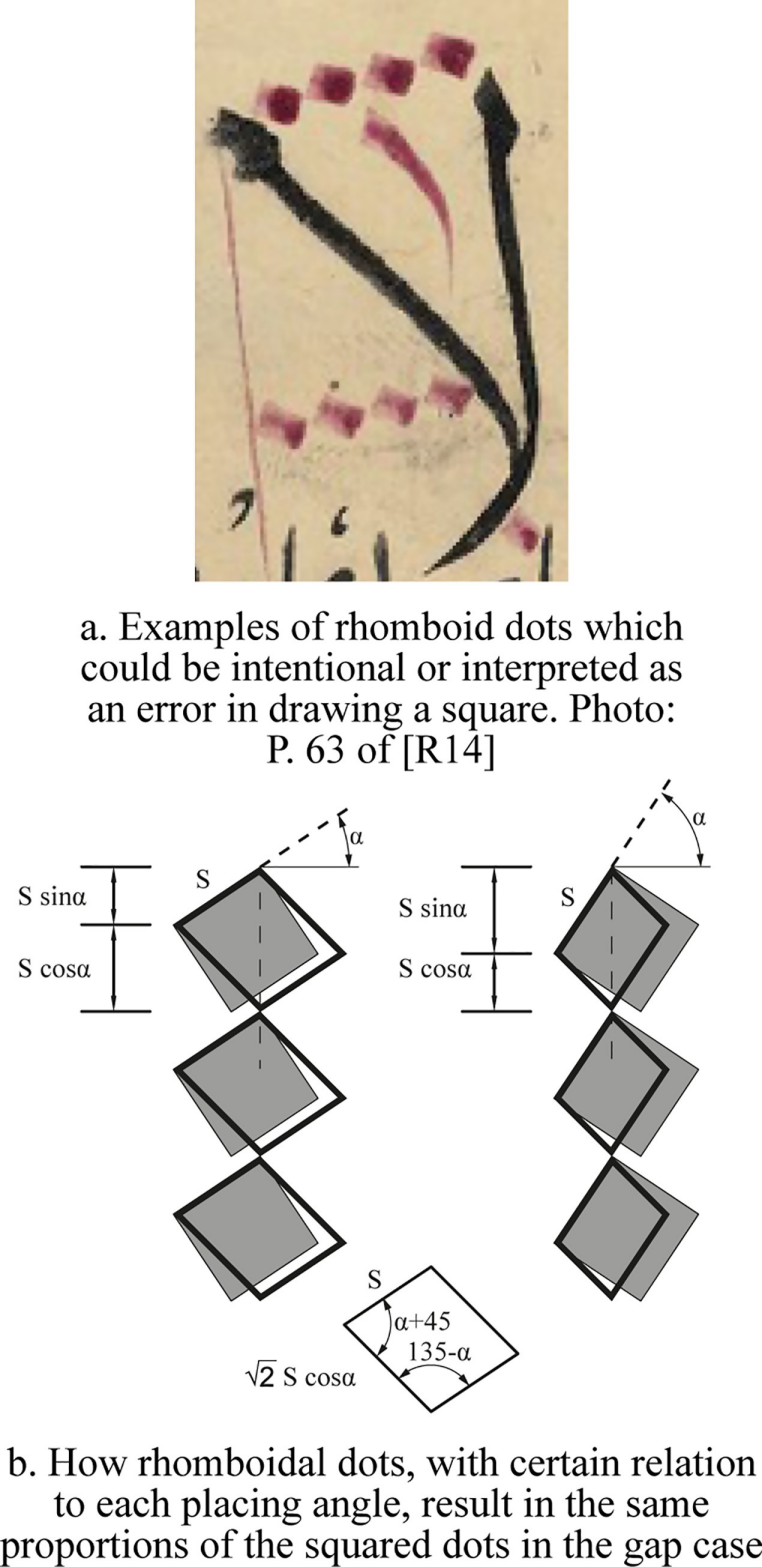
The rhomboid case.

As stated earlier, Abbot and Blair suggested that the dots are rhomboids placed vertex to vertex [9, p. 35; and 10, p. 159]. Although the dot shape in some historical manuscripts, if seen as rhomboid, can be interpreted as a square drawn in a fast and repetitive manner; a proper rhomboid can work just exactly as the squared dot in the gap case, but without leaving any gap. One of its sides should equal the squared dot side, and the other one should equal to: $\left(\sqrt{2\;}S\;\cos\propto \right)$. Its two angles should be equal to (∝+45°) and (135°−∝) as seen in Fig 13. This would work exactly as the squared dot because the total vertical distance it gives is (*S* cos ∝+*S* sin ∝), which is that of the squared dot in the gap case.

ii. The rectangle case

**Fig 14:**
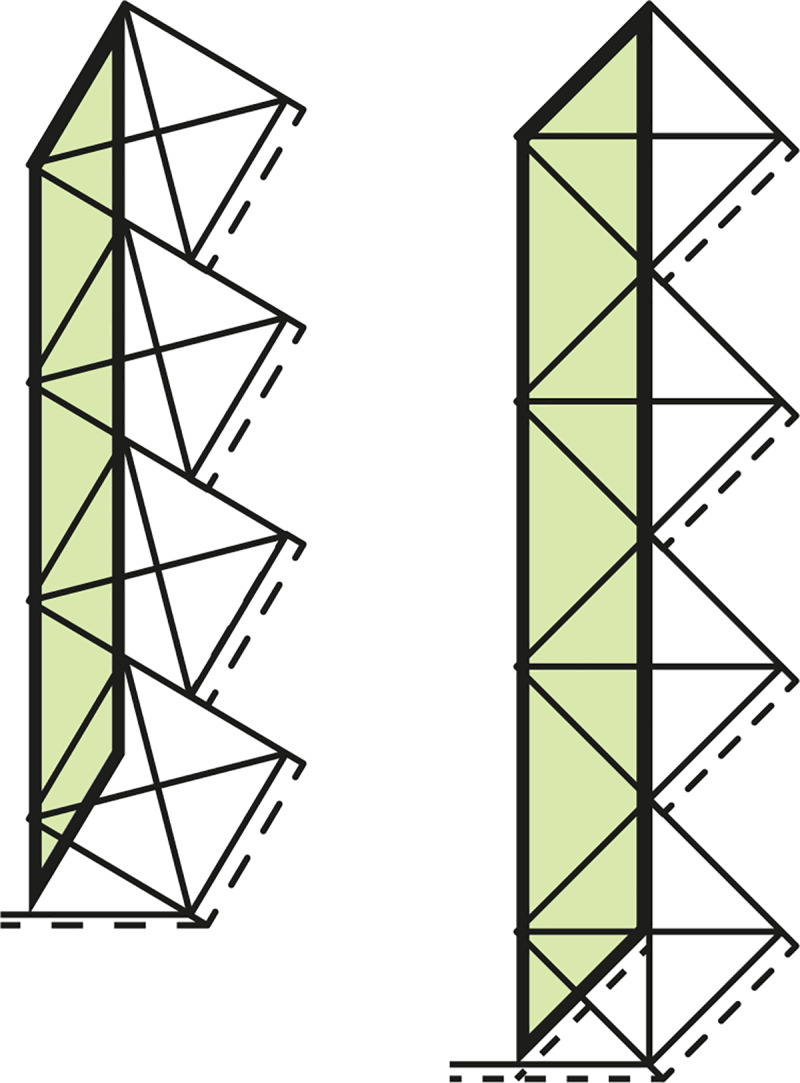
Elongated dots (proposed by some contemporary calligraphers) with partial line-connection that could lead to a very close proportional ratio.

As stated earlier, a modern calligrapher suggested a rectangular shape for the dot. If the dots were placed according to the edge case system then the final results will only differ slightly and they could really approximate the actual accurate results (Fig 14).

6 The inaccuracy of al-Qalaqshandī and al-Rāwandī‘s statements regarding the ʾAlif being the outcome of a number of vertically arranged dots, with proportions of:

(dot-width): (dots-accumulative-lengths being a whole number)

**Fig 15:**
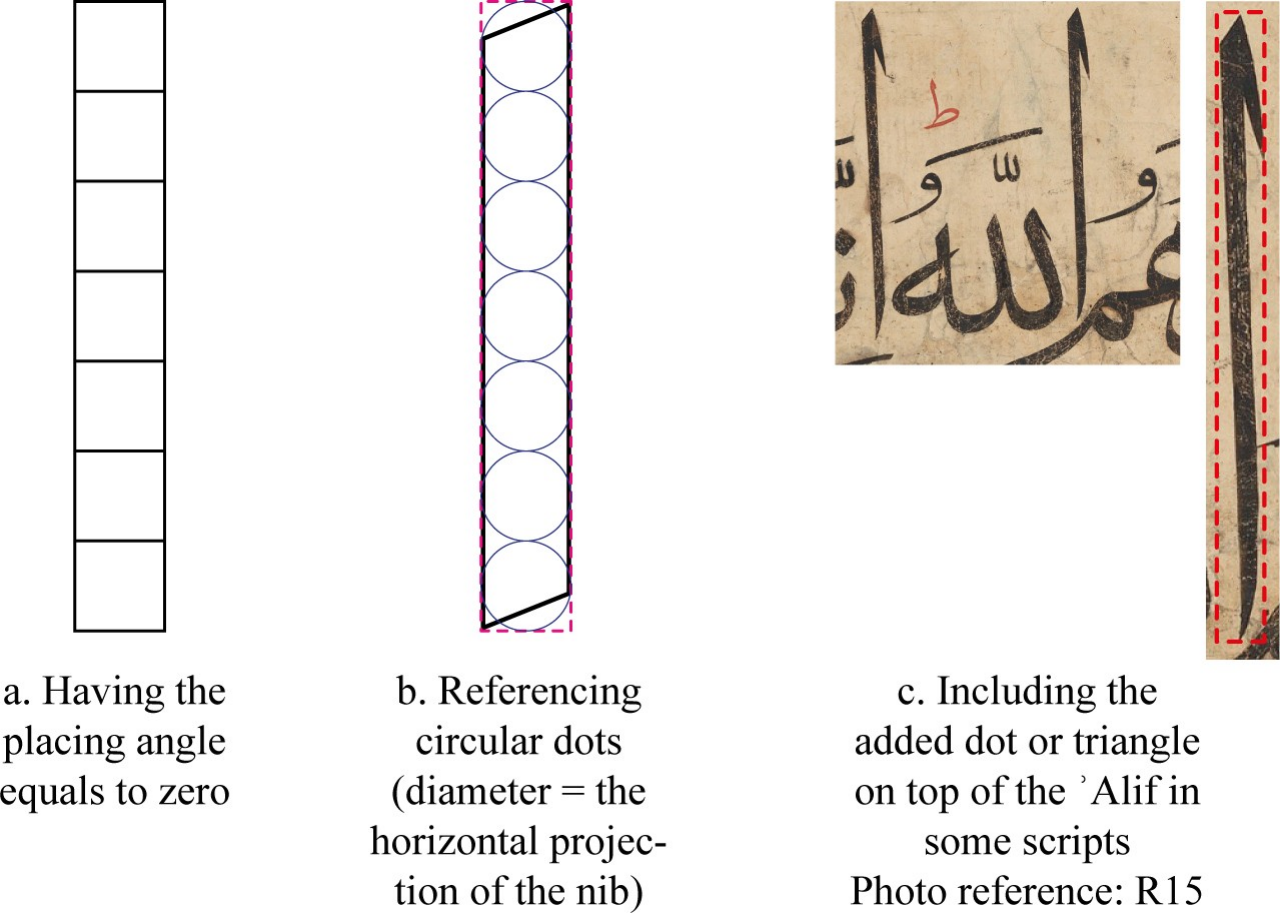
Possible interpretations of the questionable statements of al-Qalaqshandī and al-Rāwandī.

As pointed out earlier this concept works when the placing angle is equal to zero, and the historical context indicates that it was not the case except for a few instances in the Kufi script. The other two possibilities to interpret the statements of both writers so as not to consider them mistaking the concept of proportions is as follows:

i. To propose that they were talking about the circular dot of the second size presented in the dots section, and whose diameter is equal to the horizontal projection of the pen nib (Fig 4C). In this case, the dots will compose the ʾAlif whose length will be a whole number repetition of its width and is equal to the number of the dots (Fig 15B). However, al-Qalqashandī stressed, by quoting another calligrapher called Abd al-Slām, that the dot is a square in shape [7, p. 28].

ii. In some calligraphic styles, calligraphers would add a dot or a triangle at the top of the ʾAlif (Fig 15C). In such cases, the width of the ʾAlif would be that of the dot at the top, and while its body would be much slimmer the overall ʾAlif proportions will be (dot-width: sum-of-all-dot-lengths). This can satisfy the statement of al-Rāwandī who explained the existence of such a dot at the top of the ʾAlif in two scripts in the same context [18, p. 608].

These interpretations, Ikhwān al-Ṣafāʼs earlier opinion that what people are accustomed for and what writers prefer can be different from what is proposed by the rules of geometry and the virtuous ratios, and our point three of this chapter might help explain such a misconception.

## A parametric tool

**Fig 16:**
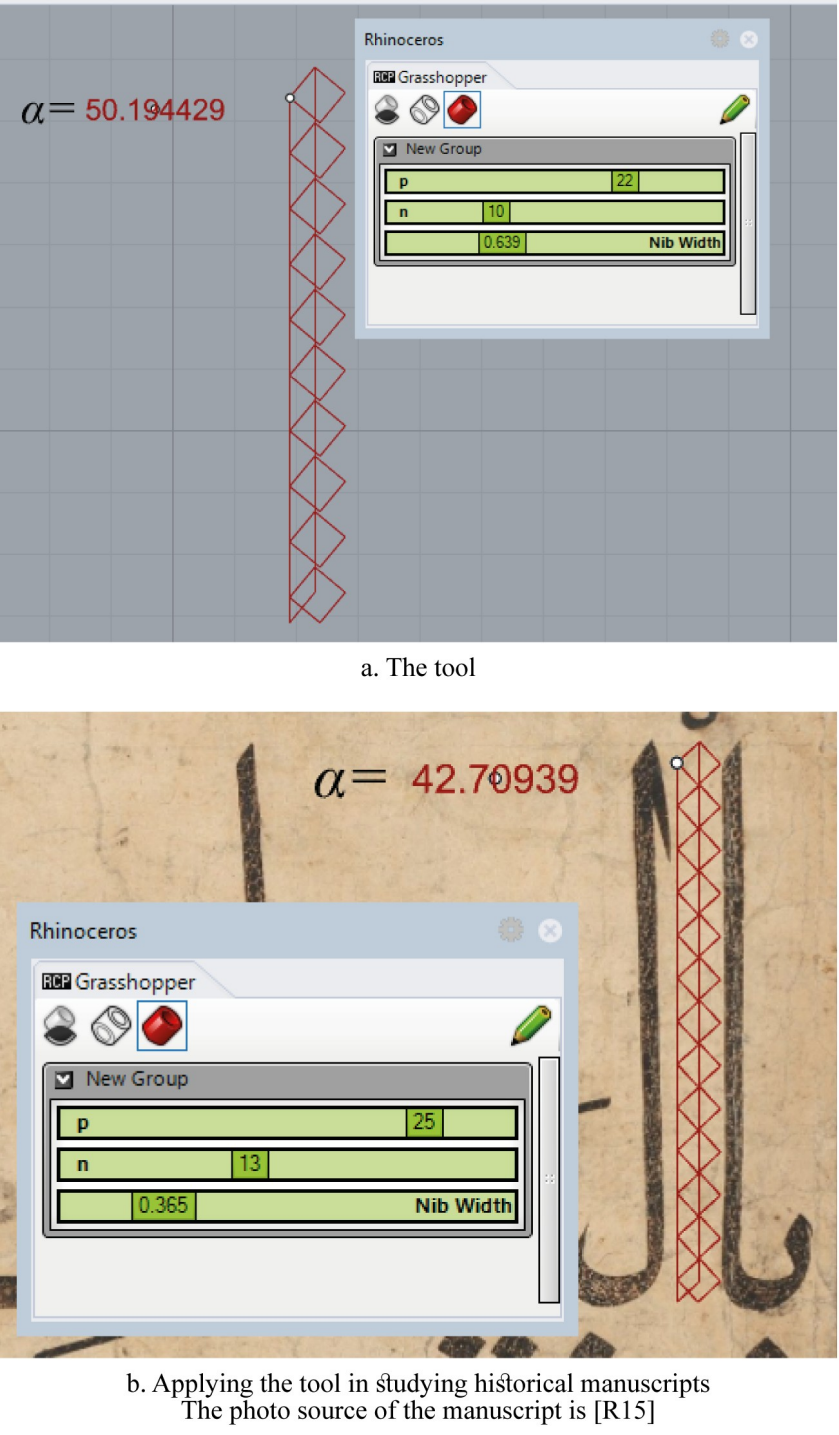
Parametric ʾAlif-creation tool.

According to the above analysis, an ʾAlif-creation parametric tool was created by the author in the Grasshopper-Rhinoceros environment. The proportions *P*, the number of points *n*, and the nib-width are the basic three variables that can be controlled. The shape of the ʾAlif and the dots are drawn, while the resulting α is indicated (Fig 16A).

Such a tool can be helpful for later studies that aim at analyzing the calligraphy of historical manuscripts (Fig 16B).

## Relating the ʾAlif to other letters

Although this paper aimed at studying the proportions of the letter ʾAlif as the basic component of the theory of proportions in Arabic calligraphy and not the whole letter set; this section will provide a short description of how it was related to other letters.

**Fig 17:**
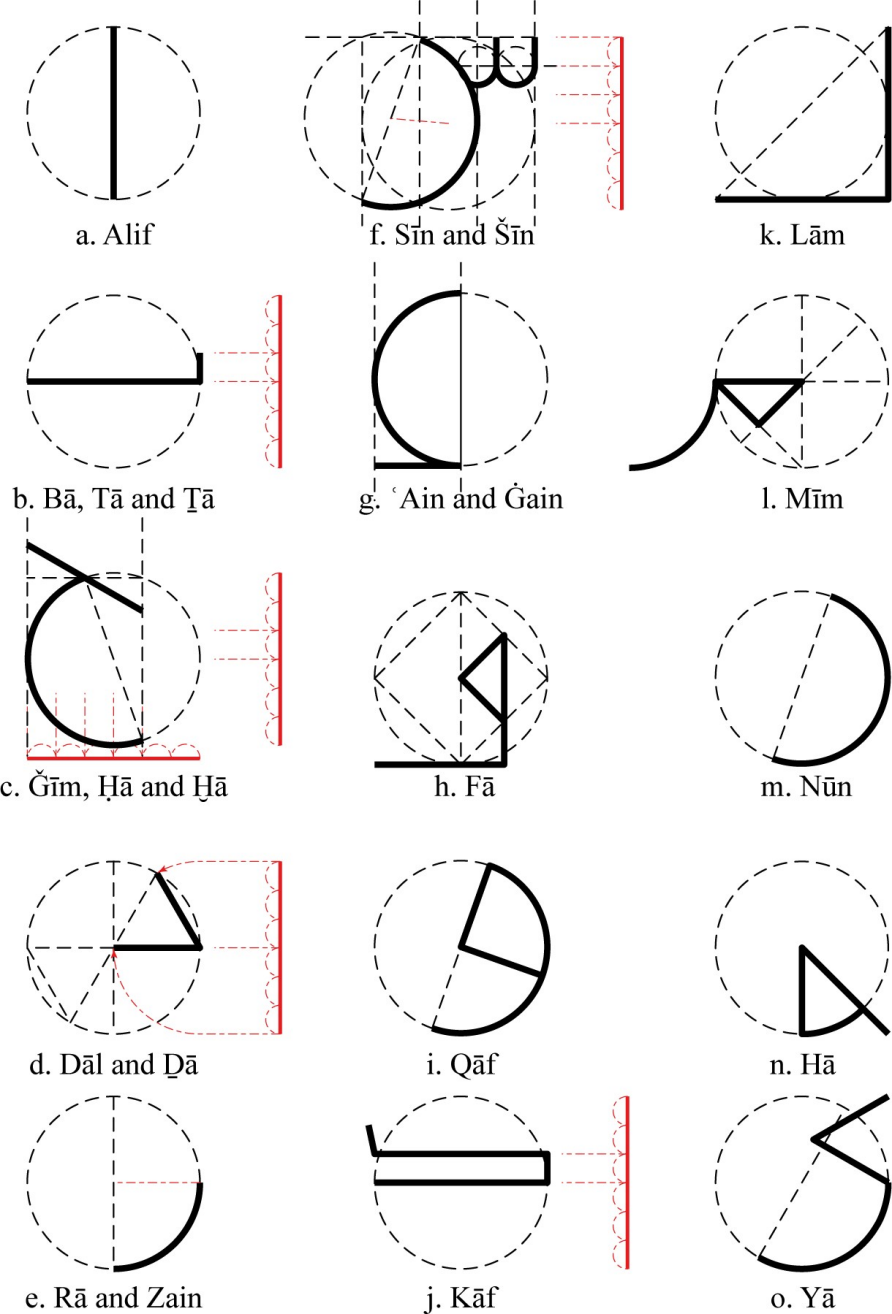
Relating the ʾAlif to other letters according to the theory (other interpretations are also possible due to the uncertainty of the text).

According to the literature that is attributed to Ibn Muqla [7, pp. 27–38; and 6], all other letters can be derived from the ʾAlif and the circle whose diameter is the ʾAlif (Fig 17A). Half, third, and sixth of the ʾAlif are used to determine the lengths of the straight parts of other letters, while half and a quarter of the circle are used to determine the curved parts. Fig 17 shows some possible visual interpretations of the textual theory.

Later references and modern manuals relate all letters to dots and their parts (halves, and horizontal and vertical projections as illustrated earlier in Figs 4 and 5) though they start with the ʾAlif and define it as the reference of all letters (modern manuals mentioned in the reference list).

The mathematical aspects of these relations must be the subject of future studies.

## Conclusion

This study analyzed the basic component of the theory of proportions in Arabic calligraphy; the letter ʾAlif, historically and mathematically.

It defined, analyzed the most important aspects of the theory, namely the letter ʾAlif, the dot, and how to relate them. It discussed all their possible interpretations, and how they might have changed over time.

It represented the possible results both as values and visually, made comparisons, explained misunderstandings and different views.

The study recommends further study to cover all other letters and details of the theory.

## Footnotes

1. Ibn Muqla, as seen in the [6] or in [7] did not refer to the dot as the measuring unit, but as the tool to differentiate between similar letter, and he proportioned all letters to the ʾAlif and its circle, all later writers, however, did refer to the dot.
2. His opinion might be supported by the work of al-Sarraj (d.928) who did mention that the origin of the letters’ forms come from a line, a circle and part of a circle combined together, but did not mention how this is actually applied [4, p. 18–19].
3. Faḥl [21, p. 33], stated that it is a rectangle for the thuluth script, and presented a figure of the dot showing an increase of about 15% in the length to the width.

## Abbreviations and photos’ credits

MET, Metropolitan Museum of Arts

Grasshopper and Rhinoceros software are trademarks of Robert McNeel and Associates.

**Table:** 

Ref.	Credit
[R^1^]	MET: Gift of Rudolf M. Riefstahl, 1930, A.No. 30.45
[R^2^]	MET: Purchase, Friends of Islamic Art Gifts, 2004, A. No. 2004.268
[R^3^]	MET: Rogers Fund, 1942, A. No. 42.63
[R^4^]	MET: Fletcher Fund, 1924, A. No. 24.146.1
[R^5^]	Ann Arbor, University of Michigan, Special Collections Library, Isl. Ms. 401 [23] https://babel.hathitrust.org/cgi/pt?id=mdp.39015088423010;view=1up;seq=1 Public Domain http://www.hathitrust.org/access_use#pd
[R^6^]	MET: Gift of Adrienne Minassian, in memory of Dr. Richard Ettinghausen, 1979, A. No.1979.201
[R^7^]	MET: Purchase, Lila Acheson Wallace Gift, 2004, A. No. 2004.87
[R^8^]	By Anonymous - https://www.nytimes.com/2015/07/23/world/europe/quran-fragments-university-birmingham.html?_r=0, Public Domain, https://commons.wikimedia.org/w/index.php?curid=41768466
[R^9^]	MET: Purchase, Lila Acheson Wallace Gift, 2004, A. No.2004.88
[R^10^]	MET: Gift of Philip Hofer, 1937, A. No. 37.142
[R^11^]	MET: Purchase, Harris Brisbane Dick Fund, funds from various donors and Dodge Fund, 2004, A. No. 2004.89
[R^12^]	MET: Fletcher Fund, 1975, A. No. 1975.201
[R^13^]	MET: Purchase, Louis E. and Theresa S. Seley Purchase Fund for Islamic Art and A. Robert Towbin Gift, 2008, A. No. 2008.31
[R^14^]	"Harvard Library, © [2019] President and Fellows of Harvard College, licensed under a Creative Commons Attribution 4.0 International License" http://id.lib.harvard.edu/aleph/011379400/catalog Record ID: 011379400 p. 29 [22]
[R^15^]	MET: Anonymous Gift, 1972, A. No. 1972.279
